# Comparison of the initial and residual speed of *Amblyomma americanum* kill on dogs treated with a single dose of Bravecto® Chew (25 mg/kg fluralaner) or Simparica TRIO® (1.2 mg/kg sarolaner, 24 µg/kg moxidectin, 5 mg/kg pyrantel)

**DOI:** 10.1186/s13071-024-06600-2

**Published:** 2025-03-21

**Authors:** Kathryn E. Reif, Michael W. Dryden, Dorothy M. Normile, Qing Kang, Brian H. Herrin, Jeba R. J. Jesudoss Chelladurai, Naemi P. Bickmeier, Cameron J. Sutherland, Mallory S. Beltz

**Affiliations:** 1 Department of Pathobiology, College of Veterinary Medicine, Auburn University, Auburn, AL 36848 USA; 2 Department of Diagnostic Medicine/Pathobiology, College of Veterinary Medicine, Kansas State University, Manhattan, KS 66506 USA; 3 Merck Animal Health, Rahway, NJ 07065 USA; 4 Department of Statistics, Kansas State University, Manhattan, KS 66506 USA

**Keywords:** Acaricide, Lone star tick, Canine, Ectoparasiticide, Isoxazoline, Prevention, Speed of kill, Tick, Tick control

## Abstract

**Background:**

To manage tick infestations and reduce tick-borne pathogen transmission risk to dogs, compliant administration of a fast-acting ectoparasiticide is necessary. Isoxazoline-containing ectoparasiticide products provide systemic whole-body coverage; however, differences in tick kill have been observed between products and these differences may be more pronounced when controlling common dose-limiting tick species such as *Amblyomma americanum*.

**Methods:**

Dogs were ranked by tick carrying capacity, randomly allocated to one of three treatment groups, and administered Bravecto® Chews (minimum 25 mg/kg fluralaner), Simparica TRIO® (minimum 1.2 mg/kg sarolaner, 24 µg/kg moxidectin, 5 mg/kg pyrantel), or no treatment. Dogs were infested with approximately 50 unfed adult (25 female, 25 male) *A. americanum* on days −2, 21, 28, and 35. Live tick counts were performed at 8, 12, 24, 48, and 72 h post-treatment (day 0) and post-infestation on days 21, 28, and 35. At each tick count timepoint, product efficacy was determined by comparing geometric mean live tick counts for each product-treated group to the untreated group and a linear mixed model was used for between-group comparisons.

**Results:**

Compared with untreated dogs, significant control of existing *A. americanum* infestations began by 8 h post-treatment (81.6%) and reached 98.0% control by 12-h for Bravecto®-treated dogs. In comparison, significant control for Simparica TRIO®-treated dogs began by 24 h post-treatment (97.7%). When reinfested on day 21, *A. americanum* infestations were controlled more quickly for Bravecto® compared with Simparica TRIO®-treated dogs at 12 h (efficacy 95.3% versus 25.5%, *P < *0.001) and 24 h (efficacy 99.7% versus 70.9%, *P* < 0.001) post-infestation. Similarly, when reinfested on day 28, faster *A. americanum* control occurred for Bravecto® compared with Simparica TRIO®-treated dogs at 12 h (efficacy 87.9% versus 18.3%, *P* < 0.001) and at 24 h (99.2% versus 59.3%, *P* < 0.001) post-infestation. Finally, when reinfested on day 35, time to ≥ 90% efficacy was achieved by 48 h for Bravecto®-treated dogs compared with 72 h post-infestation for Simparica TRIO®-treated dogs. Both products performed within label indications and no treatment-related adverse reactions occurred during the study.

**Conclusions:**

*Amblyomma americanum* infestations are controlled more quickly immediately upon treatment and at 21, 28, and 35 days post-treatment for Bravecto® compared with Simparica TRIO®-treated dogs.

**Graphical Abstract:**

**Figure:**
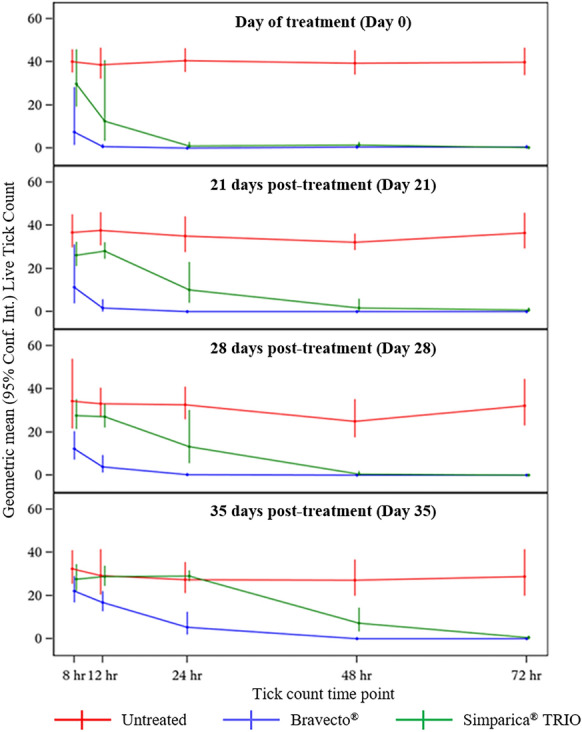

## Background

The lone star tick, *Amblyomma americanum*, is a competent vector for numerous zoonotic disease-causing agents in the USA, including *Ehrlichia chaffeensis*, *Ehrlichia ewingii*, spotted fever group *Rickettsia* spp., and probably Heartland and Bourbon viruses [1–4]. In the southeast and central USA, *A. americanum* is often the most common tick species encountered or recovered from dogs; however, this tick species is rapidly expanding northward and westward, with well-established populations reported in the northeast and Great Lakes regions [3]. A recent publication has also provided evidence that *A. americanum* likely existed much farther north of its assumed original natural range and therefore may actually be reclaiming its former natural range [5].

The considerable geographic range of this aggressive, vector-competent tick with a wide host-range makes companion animals, especially dogs, vulnerable to infestation and possible infection with numerous disease-causing pathogens [1, 2]. Effective tick control, ideally provided year-round and administered as instructed, offer dogs and cats the best protection from tick infestation and tick-borne diseases.

*Amblyomma americanum* is considered one of the more difficult species of ticks to control with isoxazoline-containing ectoparasiticides. Reflecting the hardiness of this tick species, most isoxazoline-containing ectoparasiticides have a label indication for ≥ 90% effectiveness against *A. americanum* 72 h immediately post-treatment and post-reinfestation compared with a 48-h label efficacy indication for other common US tick species such as *Ixodes scapularis*, *Dermacentor variabilis*, and *Rhipicephalus sanguineus* (sensu lato). Previous speed of tick kill studies using isoxazoline drugs have been investigated for *A. americanum*. For example, the efficacy of sarolaner (Simparica®) administered orally to dogs at 2–4 mg/kg against adult *A. americanum* (based on geometric means) at 24 h post-infestation on day 28 was 83.1% [6]. In the same study, the efficacy of fluralaner (Bravecto®) administered orally to dogs at 25–56 mg/kg was 82.4% at the same timepoint [6]. In another study, the efficacy of afoxolaner (2.5–6.8 mg/kg) against *A. americanum* 24 h post-infestation 28 days post-treatment was 41.5% (based on geometric means) [7]. In that same study the efficacy of sarolaner (2–4 mg/kg) was 91.1% at the same timepoint [7].

Recently, it was demonstrated that the reduced dosage of sarolaner in Simparica TRIO® (minimal sarolaner dose 1.2 mg/kg in Simparica TRIO®) resulted in a slower residual speed of tick kill compared with the dose of sarolaner in Simparica® (minimal sarolaner dose 2.0 mg/kg in Simparica®) [8, 9]. The two sarolaner dosages were investigated for their speed to control *Ixodes scapularis*, with dogs in the first study treated with Simparica® and dogs in the second study treated with Simparica TRIO®. In the study evaluating Simparica®, when dogs were reinfested at 21 days post-treatment, efficacy of *I. scapularis* control at 8 and 12 h post-infestation was 33.5% and 68.8%, respectively (based on geometric means) [8]. In comparison, in the Simparica TRIO® study, the efficacy at these same timepoints was 0% and 39.4%, respectively (based on geometric means) [9]. These data collectively support that the reduced amount of sarolaner in Simparica TRIO® compared with the amount of sarolaner in the stand-alone oral Simparica® product negatively impacts speed of tick kill.

The objective of this study was to compare the initial and residual speed of tick kill provided by a single oral dose of fluralaner (Bravecto® Chews) or sarolaner (combined with moxidectin and pyrantel, Simparica TRIO®) against *A. americanum*-infested dogs.

## Methods

### Compliance

This study was conducted in accordance with the guidelines for evaluating the efficacy of canine and feline parasiticides for the treatment, prevention, and control of flea and tick infestations set forth by the World Association for the Advancement of Veterinary Parasitology (WAAVP) [10] and the Good Clinical Practice document that provides guidance on the design and conduct of all clinical studies of veterinary products in the target species [11].

This study was conducted as a masked, randomized, complete block design positive- and negative-controlled laboratory efficacy study. Personnel involved in animal observations, infestation procedures, tick count, and live–dead assessment procedures were masked to the treatment groups. As previously described, personal protective equipment was worn by all personnel conducting tick infestations and tick counts to avoid skin contact with dogs and changed to ensure no possible transfer of any active ingredient when working with dogs in different groups [9]. Personnel that participated in treatment administration were not involved in conducting or recording tick counts, tick live–dead assessments, or dog health assessments. All animal work was conducted in full compliance with an approved Institutional Animal Care and Use Committee Protocol (IACUC Protocol 4677) on file with the University Research Compliance Office at Kansas State University (KSU) in Manhattan, KS.

### Animals & allocation

A total of 24 purpose-bred beagles (> 6 months of age, 5.0–7.5 kg, 12F:12M) were enrolled in the study. All enrolled dogs met inclusion criteria: be clinically healthy with no preexisting conditions; greater or equal to 6 months of age; demonstrated susceptibility to tick infestation (> 25% recovery of live ticks from test infestation); amenable temperaments for handling; and have not been treated with an anti-tick or anti-flea products for the past 180 days. On the basis of descending live tick count, enrolled dogs were ranked and blocked into eight blocks of three dogs. Within each block, dogs were randomly assigned to one of three groups using the randomization function on a spreadsheet program (Microsoft Excel 2019, Redmond, WA). Dogs were housed individually in concrete runs that had solid side walls so that they could not physically contact each other. Dogs were fed a maintenance ration once daily and provided water ad libitum. General health observations were performed at least once daily for all dogs.

### Tick infestations

Tick infestations were performed using laboratory-reared, adult *Amblyomma americanum* approximately 12 weeks post-molt (Ecto Services, LLC, Henderson, NC). Ticks were maintained in Dr. Kathryn Reif’s laboratory at Kansas State University for up to 2 weeks at room temperature and 92–94% relative humidity until dog infestation. Live ticks were sorted and counted into vials (50 ticks/vial) the day prior to dog infestation.

Tick infestations were conducted on dogs on days −2, 21, 28, and 35. At each infestation timepoint, each dog was infested with approximately 50 unfed adult *A. americanum* (25 female, 25 male). As in a previous study, infestations were conducted on non-sedated dogs and ticks were applied directly to the dog’s fur along dorsal side of the head, neck, and back [9]. Dogs were transferred to pet carriers within 5 min of the tick infestation and maintained in the carrier for 4 h to facilitate tick attachment. Following this 4-h period, dogs were removed from the carrier and returned to their individual pens. Ticks that failed to infest dogs were collected from pet carriers and disposed (not included in counts or analyses).

### Treatments

On treatment day (day 0), dogs in group 1 (untreated) received no treatment; each dog in group 2 received a fluralaner chewable tablet (Bravecto® Chew, Merck Animal Health, Rahway, NJ, USA) on the basis of the dog’s individual body weight to achieve a minimum dose of 25 mg/kg fluralaner; and each dog in group 3 received a sarolaner-moxidectin-pyrantel chewable tablet (Simparica TRIO®, Zoetis Animal Health, Parsippany-Troy Hills, NJ) on the basis of the dog’s individual body weight to achieve a minimum dose of 1.2 mg/kg sarolaner, 24 µg/kg moxidectin, and 5.0 mg/kg pyrantel. All treatments were provided with a small spoonful of wet dog food approximately 30 min after the dogs’ morning meal. Untreated dogs were similarly handled but received only a small spoonful of wet dog food. Dogs were observed to assess whether the chewable tablets were spit or vomited out and monitored for adverse treatment reaction signs at 1, 2, and 4 h post-treatment. No dog experienced any adverse treatment reaction nor spat out or vomited the chewable tablet.

### Tick counts

Tick assessment and counting procedures were performed as previously described [8]. Tick counts for initial speed of tick kill analysis were conducted at 8, 12, 24, 48, and 72 h (± 0.25-h) post-treatment (day 0). Residual speed of tick kill was assessed by conducting tick counts at 8, 12, 24, 48, and 72 h (± 0.25 h), post-infestation on days 21, 28, and 35. Ticks were left in situ on dogs following the 8-, 12-, 24-, and 48-h counts, but were removed with forceps after the 72-h tick count. Sex (male or female), attachment status (unattached or attached), and viability status (live or dead) was determined for each observed tick [8, 9].

### Statistical analysis

Counts of live ticks were analyzed separately for each timepoint of every post-enrollment infestation using a linear mixed model. Treatment group was the fixed effect and block was the random effect. Error term variance was taken as heterogeneous with respect to treatment group. For efficacy assessment based on geometric means, counts of live ticks were subjected to $\ln ({\text{count}} + 1)$ transformation before statistical modeling. Denote ${\text{LSM}}_{\ln } (i)$ as the corresponding least squares mean of treatment group *i*, and efficacy of treatment group *i* relative to treatment group *j* was calculated as

$$1 - \frac{{\exp ({\text{LSM}}_{\ln } (i)) - 1}}{{\exp ({\text{LSM}}_{\ln } (j)) - 1}},$$

where exp(LSM_ln_(*i*))  − 1 represents the model-based estimate of geometric mean for treatment group *i*. For efficacy assessment based on arithmetic means, counts of live ticks were modeled directly without any transformation. Denote ${\text{LSM}}(i)$ as the corresponding least squares mean of treatment group *i*. Efficacy of treatment group *i* relative to treatment group *j* was calculated as

$$1 - \frac{{{\text{LSM}}(i)}}{{{\text{LSM}}(j)}},$$

where ${\text{LSM}}(i)$ represents the model-based estimate of arithmetic mean for treatment group *i*. To assure convergence, variances for block and error term were bounded low by 10^–3^ for transformed counts and 10^–1^ for untransformed counts.

A tick-free dog was defined as a dog with no live ticks [i.e., an infested dog has one or more live tick(s)]. The percentage of tick-free dogs by treatment group was analyzed using the Fisher’s exact test for treatment group comparison at a given time when both groups were not 100% infested.

All tests were conducted at the 0.05 significance level. Pairwise comparisons were carried out using two-sided tests. No multiplicity adjustment was performed.

Statistical analyses were performed using Statistical Analysis Software (SAS version 9.4; Cary, NC) PROC MIXED.

## Results

### Dogs removed from study

Two dogs in the untreated control group were removed from the study. One dog (BJS-1) was removed on day 0 (treatment day) because it was inadvertently dosed with tick control product. The second untreated control group dog (AET-1) was removed because it experienced a systemic edematous reaction within 4 h following tick infestation on day 21. Marked swelling and redness occurred primarily around eyes, lips, ears, muzzle, and front feet. Heart and respiratory rate were within normal limits. This dog was taken immediately to the veterinary teaching hospital and treated with 2 mg/kg diphenhydramine via subcutaneous injection. The dog responded rapidly (within 10 min) to the antihistamine and was bright and alert. By 30 min post-treatment, swelling was reduced over 50%. This dog was removed from the study, all remaining ticks were manually removed, and this dog was treated with an acaricide. Therefore, there are seven dogs in the untreated control group for day 0 initial speed of tick kill data and six dogs in the untreated control group for all residual speed of tick kill datasets for days 21, 28, and 35.

### Product dosing and safety

Overall, dogs averaged 6.1 kg (treatment group 1: 6.2 kg, treatment group 2: 5.8 kg, treatment group 3: 6.4 kg). Dogs in treatment group 2 received an average of 43.7 mg/kg fluralaner (range 37.8–49.2 mg/kg). Dogs in treatment group 3 received an average of 1.89 mg/kg sarolaner (range 1.61–2.04 mg/kg). No dog in either product-treated group had a treatment-related adverse event at the time of or within 4 h of treatment. No dog spit the chew out or required re-dosing.

One dog (XLT-1) in the Simparica TRIO® treatment group developed a mild allergic response (mild swelling and redness around eyes and muzzle) after the fourth tick infestation on day 28 post-treatment. This reaction was similar, but less severe, to the one previously described for dog BJS-1, the dog removed from the untreated control group. Dog XLT-1 was bright and alert. Heart and respiratory rate were within normal limits. The allergic reaction was noted 4 h post-infestation when the dog was removed from its infestation crate. This dog was treated with oral diphenhydramine (25 mg/kg) and the swelling resolved within a few hours of treatment. This dog remained enrolled in the study. When infested for the fifth time on day 35, the dog was watched closely and did not experience a similar allergic response to this final tick infestation.

No product-associated serious adverse events were observed during this study. Three dogs in the untreated group and one dog in the Simparica TRIO® treatment group each had an observation of soft stools/diarrhea of 24–48-h duration that resolved without treatment intervention.

### Tick counts and product efficacy

Summary statistics for live tick counts are presented in Table 1. Tick control efficacy results presented and discussed are based on analyses using geometric means of live tick counts; however, data and analyses using both geometric and arithmetic means are provided in Table 2.

**Table 1:** Summary statistics for *Amblyomma americanum* on dogs treated with Bravecto® or Simparica TRIO®

Tick count time^1^	Treatment group	Day 0^*^ (treatment day)					Day 21 infestation					Day 28 infestation					Day 35 infestation				
		*N*	# of live ticks			#/# (%) tick-free dogs	*N*	# of live ticks			#/# (%) tick-free dogs	*N*	# of live ticks			#/# (%) tick-free dogs	*N*	# of live ticks			#/# (%) tick-free dogs
			Mean^2^	Min	Max			Mean^2^	Min	Max			Mean^2^	Min	Max			Mean^2^	Min	Max	
8 h	Untreated control	7	40.5	32	52	0/7 (0%)	6	35.2	27	44	0/6 (0%)	6	32.7	22	46	0/6 (0%)	6	32.2	17	40	0/6 (0%)
	Bravecto®	8	15.5	0	38	2/8 (25%)	8	16.3	1	31	0/8 (0%)	8	14.6	6	31	0/8 (0%)	8	23	14	32	0/8 (0%)
	Simparica TRIO®	8	33.1	8	47	0/8 (0%)	8	27.3	16	38	0/8 (0%)	8	28.8	16	43	0/8 (0%)	8	28.5	15	36	0/8 (0%)
12 h	Untreated control	7	39.4	28	55	0/7 (0%)	6	37.2	29	46	0/6 (0%)	6	32	24	40	0/6 (0%)	6	30.2	18	44	0/6 (0%)
	Bravecto®	8	1.1	0	3	4/8 (50%)	8	3.4	0	12	3/8 (37.5%)	8	6	1	22	0/8 (0%)	8	17.6	9	29	0/8 (0%)
	Simparica TRIO®	8	21.4	0	43	1/8 (12.5%)	8	28.4	22	37	0/8 (0%)	8	28	16	42	0/8 (0%)	8	29.3	21	37	0/8 (0%)
24 h	Untreated control	7	40.9	31	51	0/7 (0%)	6	35.7	24	46	0/6 (0%)	6	33.3	24	44	0/6 (0%)	6	27.7	19	41	0/6 (0%)
	Bravecto®	8	0.1	0	1	7/8 (87.5%)	8	0.1	0	1	7/8 (87.5%)	8	0.4	0	2	6/8 (75%)	8	7.3	0	13	1/8 (12.5%)
	Simparica TRIO®	8	1.8	0	7	4/8 (50%)	8	14	1	29	0/8 (0%)	8	17.4	1	26	0/8 (0%)	8	29.1	25	35	0/8 (0%)
48 h	Untreated control	7	39	30	47	0/7 (0%)	6	32.5	28	39	0/6 (0%)	6	26.2	17	38	0/6 (0%)	6	28	16	37	0/6 (0%)
	Bravecto®	8	0.8	0	4	5/8 (62.5%)	8	0.1	0	1	7/8 (87.5%)	8	0.1	0	1	7/8 (87.5%)	8	0.3	0	2	7/8 (87.5%)
	Simparica TRIO®	8	1.8	0	4	2/8 (25%)	8	4.1	0	15	4/8 (50%)	8	1.1	0	5	5/8 (62.5%)	8	9.5	2	21	0/8 (0%)
72 h	Untreated control	7	40.3	32	49	0/7 (0%)	6	37.3	30	54	0/6 (0%)	6	33.3	18	43	0/6 (0%)	6	30.2	18	43	0/6 (0%)
	Bravecto®	8	0.9	0	3	4/8 (50%)	8	0	0	0	8/8 (100%)	8	0	0	0	8/8 (100%)	8	0.3	0	1	6/8 (75%)
	Simparica TRIO®	8	0.4	0	1	5/8 (62.5%)	8	1.1	0	3	4/8 (50%)	8	0.3	0	1	6/8 (75%)	8	0.9	0	3	4/8 (50%)

^*^Dogs were infested on day −2 and tick counts were performed upon treatment (day 0)

^1^Tick count time post-treatment (day 0) or post-reinfestation (days 21, 28, or 35)

^2^Geometric mean

**Table 2:** Efficacy of Bravecto® and Simparica TRIO® to control adult *Amblyomma americanum* infestation upon treatment (day 0) and at 21, 28, and 35 days post-treatment

Infestation day^1^	Tick count time^2^	Treatment group	Model ln(count + 1)					Model count directly				
			Geometric Mean ± SE	Compared with untreated control		Compared with Simparica TRIO®		Arithmetic Mean ± SE	Compared with untreated control		Compared with Simparica TRIO®	
				Test statistic (DOF) *P*-value	Efficacy (%)^3^	Test statistic (DOF) *P*-value	Efficacy (%)^3^		Test statistic (DOF) *P*-value	Efficacy (%)^3^	Test statistic (DOF) *P*-value	Efficacy (%)^3^
Day −2 (48 h prior to treatment)	8 h	Untreated control	40.1 ± 2.2					40.5 ± 2.3				
		Bravecto®	7.4 ± 4.5	−3.0 (7.0) 0.020	81.6%	−2.3 (8.5) 0.046	75.2%	15.5 ± 6.1	−4.3 (6.2) 0.005	61.8%	−2.6 (9.5) 0.028	53.2%
		Simparica TRIO®	29.8 ± 5.6	−1.6 (7.0) 0.146	25.6%			33.1 ± 4.1	−2.0 (5.4) 0.104	18.3%		
	12 h	Untreated control	38.8 ± 2.9					39.4 ± 3.1				
		Bravecto®	0.8 ± 0.4	−13 (8.4) < 0.001	98.0%	−3.9 (10.1) 0.003	93.9%	1.1 ± 0.5	−12 (6.3) < 0.001	97.1%	−3.6 (7.1) 0.009	94.7%
		Simparica TRIO®	12.6 ± 6.5	−2.2 (7.3) 0.059	67.4%			21.4 ± 5.6	−2.8 (10.7) 0.017	45.8%		
	24 h	Untreated control	40.5 ± 2.2					40.8 ± 2.2				
		Bravecto®	0.1 ± 0.1	−36 (11.0) < 0.001	99.8%	−1.8 (8.1) 0.103	90.3%	0.1 ± 0.2	−18 (6.0) < 0.001	99.7%	−1.7 (7.1) 0.140	92.9%
		Simparica TRIO®	0.9 ± 0.6	−10 (7.4) < 0.001	97.7%			1.7 ± 1.0	−16 (8.2) < 0.001	95.7%		
	48 h	Untreated control	39.3 ± 2.2					39.2 ± 2.0				
		Bravecto®	0.5 ± 0.3	−17 (6.4) < 0.001	98.8%	−1.8 (12.7) 0.103	67.4%	0.8 ± 0.5	−19 (6.2) < 0.001	98.1%	−2.0 (7.1) 0.084	57.1%
		Simparica TRIO®	1.4 ± 0.5	−13 (7.0) < 0.001	96.4%			1.8 ± 0.5	−18 (6.3) < 0.001	95.5%		
	72 h	Untreated control	39.8 ± 2.6					40.3 ± 2.6				
		Bravecto®	0.6 ± 0.3	−15 (8.3) < 0.001	98.4%	1.0 (11.7) 0.361	0%	0.9 ± 0.4	−15 (6.2) < 0.001	97.8%	1.4 (7.4) 0.194	0%
		Simparica TRIO®	0.3 ± 0.2	−24 (10.0) < 0.001	99.3%			0.4 ± 0.2	−15 (6.0) < 0.001	99.1%		
Day 21 (21 days post-treatment)	8 h	Untreated control	36.7 ± 2.9					35.2 ± 2.5				
		Bravecto®	11.4 ± 5.0	−2.8 (6.9) 0.028	68.9%	−1.9 (7.3) 0.092	56.5%	16.3 ± 4.2	−3.9 (11.0) 0.003	53.8%	−2.2 (12.2) 0.049	40.4%
		Simparica TRIO®	26.2 ± 2.6	−4.2 (4.0) 0.014	28.5%			27.3 ± 2.8	−2.1 (11.9) 0.058	22.5%		
	12 h	Untreated control	37.6 ± 3.0					37.2 ± 2.7				
		Bravecto®	1.8 ± 1.1	−6.9 (6.9) < 0.001	95.3%	−6.2 (6.8) < 0.001	93.7%	3.4 ± 1.6	−11 (8.4) < 0.001	90.9%	−11 (13.9) < 0.001	88.1%
		Simparica TRIO®	28.0 ± 1.6	−3.5 (2.8) 0.046	25.5%			28.4 ± 1.7	−2.8 (8.8) 0.021	23.7%		
	24 h	Untreated control	35.0 ± 3.2					35.6 ± 3.0				
		Bravecto®	0.1 ± 0.1	−30 (7.8) < 0.001	99.7%	−7.0 (7.6) < 0.001	99.1%	0.1 ± 0.2	−12 (5.0) < 0.001	99.6%	−4.0 (7.0) 0.005	99.1%
		Simparica TRIO®	10.2 ± 3.6	−3.5 (7.6) 0.009	70.9%			14.0 ± 3.5	−4.7 (12.0) < 0.001	60.7%		
	48 h	Untreated control	32.1 ± 1.5					32.5 ± 1.6				
		Bravecto®	0.1 ± 0.1	−39 (7.6) < 0.001	99.7%	−2.1 (7.4) 0.068	94.6%	0.1 ± 0.2	−20 (5.0) < 0.001	99.6%	−1.8 (7.0) 0.114	97.0%
		Simparica TRIO®	1.7 ± 1.1	−6.1 (6.9) < 0.001	94.8%			4.1 ± 2.2	−10 (11.6) < 0.001	87.3%		
	72 h	Untreated control	36.6 ± 3.2					37.3 ± 3.6				
		Bravecto®	0.0 ± 0.0	−43 (5.1) < 0.001	100%	−2.5 (7.0) 0.041	100%	0.0 ± 0.2	−10 (5.0) < 0.001	100%	−2.3 (7.4) 0.052	100%
		Simparica TRIO®	0.8 ± 0.4	−13 (8.8) < 0.001	97.9%			1.1 ± 0.5	−10 (5.2) < 0.001	97.0%		
Day 28 (28 days post-treatment)	8 h	Untreated control	34.3 ± 4.1					35.6 ± 3.9				
		Bravecto®	12.2 ± 2.8	−4.9 (7.1) 0.002	64.4%	−3.9 (4.7) 0.013	55.7%	14.6 ± 4.8	−4.8 (11.3) < 0.001	58.9%	−3.8 (7.0) 0.006	49.1%
		Simparica TRIO®	27.5 ± 3.0	−2.5 (5.1) 0.056	19.7%			28.8 ± 3.1	−2.9 (5.0) 0.034	19.2%		
	12 h	Untreated control	33.2 ± 2.8					33.7 ± 2.7				
		Bravecto®	4.0 ± 1.6	−6.2 (7.0) < 0.001	87.9%	−5.5 (7.2) < 0.001	85.2%	6.0 ± 4.4	−7.3 (6.5) < 0.001	82.2%	−5.8 (7.3) < 0.001	78.6%
		Simparica TRIO®	27.1 ± 2.5	−3.8 (4.5) 0.016	18.3%			28.0 ± 2.7	−3.9 (3.1) 0.028	16.9%		
	24 h	Untreated control	32.7 ± 2.9					33.3 ± 3.0				
		Bravecto®	0.3 ± 0.2	−19 (10.6) < 0.001	99.2%	−6.7 (9.8) < 0.001	98.1%	0.4 ± 0.3	−11 (5.1) < 0.001	98.9%	−5.1 (7.1) 0.001	97.8%
		Simparica TRIO®	13.3 ± 4.7	−2.5 (7.9) 0.036	59.3%			17.4 ± 3.3	−3.6 (11.9) 0.004	47.8%		
	48 h	Untreated control	25.0 ± 3.4					26.2 ± 3.5				
		Bravecto®	0.1 ± 0.1	−20 (9.0) < 0.001	99.6%	−1.5 (8.5) 0.180	85.5%	0.1 ± 0.2	−7.3 (5.0) < 0.001	99.5%	−1.5 (7.2) 0.181	88.9%
		Simparica TRIO®	0.6 ± 0.4	−9.6 (10.0) < 0.001	97.5%			1.1 ± 0.7	−6.9 (5.3) < 0.001	95.7%		
	72 h	Untreated control	32.2 ± 4.1					33.3 ± 3.6				
		Bravecto®	0.0 ± 0.0	−28 (5.0) < 0.001	100%	−1.5 (7.1) 0.171	100%	0.0 ± 0.2	−9.2 (5.0) < 0.001	100%	−1.4 (10.7) 0.197	100%
		Simparica TRIO®	0.2 ± 0.1	−20 (11.2) < 0.001	99.4%			0.3 ± 0.2	−9.1 (5.0) < 0.001	99.2%		
Day 35^4^ (35 days post-treatment)	8 h	Untreated control	32.4 ± 3.4					33.3 ± 2.8				
		Bravecto®	22.2 ± 2.7	−4.0 (8.9) 0.003	31.5%	−2.7 (7.2) 0.032	19.9%	23.0 ± 2.6	−4.1 (8.3) 0.003	31.0%	−3.0 (7.7) 0.018	19.3%
		Simparica TRIO®	27.7 ± 2.6	−2.7 (4.7) 0.043	14.5%			28.5 ± 2.3	−2.3 (4.1) 0.081	14.5%		
	12 h	Untreated control	29.2 ± 3.9					30.3 ± 4.0				
		Bravecto®	16.9 ± 2.0	−3.2 (10.4) 0.010	42.2%	−4.2 (6.9) 0.004	41.4%	17.6 ± 2.0	−2.9 (6.5) 0.024	41.8%	−4.8 (5.1) 0.005	39.7%
		Simparica TRIO®	28.8 ± 2.0	−0.1 (6.7) 0.928	1.4%			29.3 ± 1.9	−0.2 (6.2) 0.820	3.3%		
	24 h	Untreated control	27.4 ± 2.9					28.2 ± 3.0				
		Bravecto®	5.3 ± 2.0	−4.5 (8.2) 0.002	80.6%	−4.9 (7.0) 0.002	81.7%	7.2 ± 1.7	−6.5 (6.9) < 0.001	74.3%	−13 (7.9) < 0.001	75.1%
		Simparica TRIO®	29.0 ± 1.1	0.5 (5.0) 0.614	0%			29.1 ± 1.1	0.3 (5.1) 0.770	0%		
	48 h	Untreated control	27.2 ± 3.2					28.0 ± 2.9				
		Bravecto®	0.1 ± 0.2	−19 (8.8) < 0.001	99.5%	−6.8 (9.4) < 0.001	98.0%	0.2 ± 0.3	−9.4 (5.0) < 0.001	99.1%	−3.7 (7.1) 0.008	97.4%
		Simparica TRIO®	7.3 ± 2.2	−4.4 (7.2) 0.003	73.1%			9.5 ± 2.5	−4.8 (10.8) < 0.001	66.1%		
	72 h	Untreated control	28.8 ± 4.1					30.1 ± 4.2				
		Bravecto®	0.2 ± 0.1	−18 (10.5) < 0.001	99.3%	−1.4 (11.0) 0.202	69.6%	0.2 ± 0.2	−7.2 (5.0) < 0.001	99.2%	−1.5 (7.7) 0.168	71.4%
		Simparica TRIO®	0.6 ± 0.3	−12 (11.5) < 0.001	97.8%			0.9 ± 0.4	−7.0 (5.1) < 0.001	97.1%		

^1^Infestation day relative to treatment day (day 0)

^2^Tick count time post-treatment (day 0) or post-reinfestation (days 21, 28, or 35)

^3^Any calculated negative efficacy values are reported as 0

^4^Simparica TRIO® is a monthly administered tick control product. Evaluation of Simparica TRIO® at day 35 was for experimental purposes

**Fig. 1:**
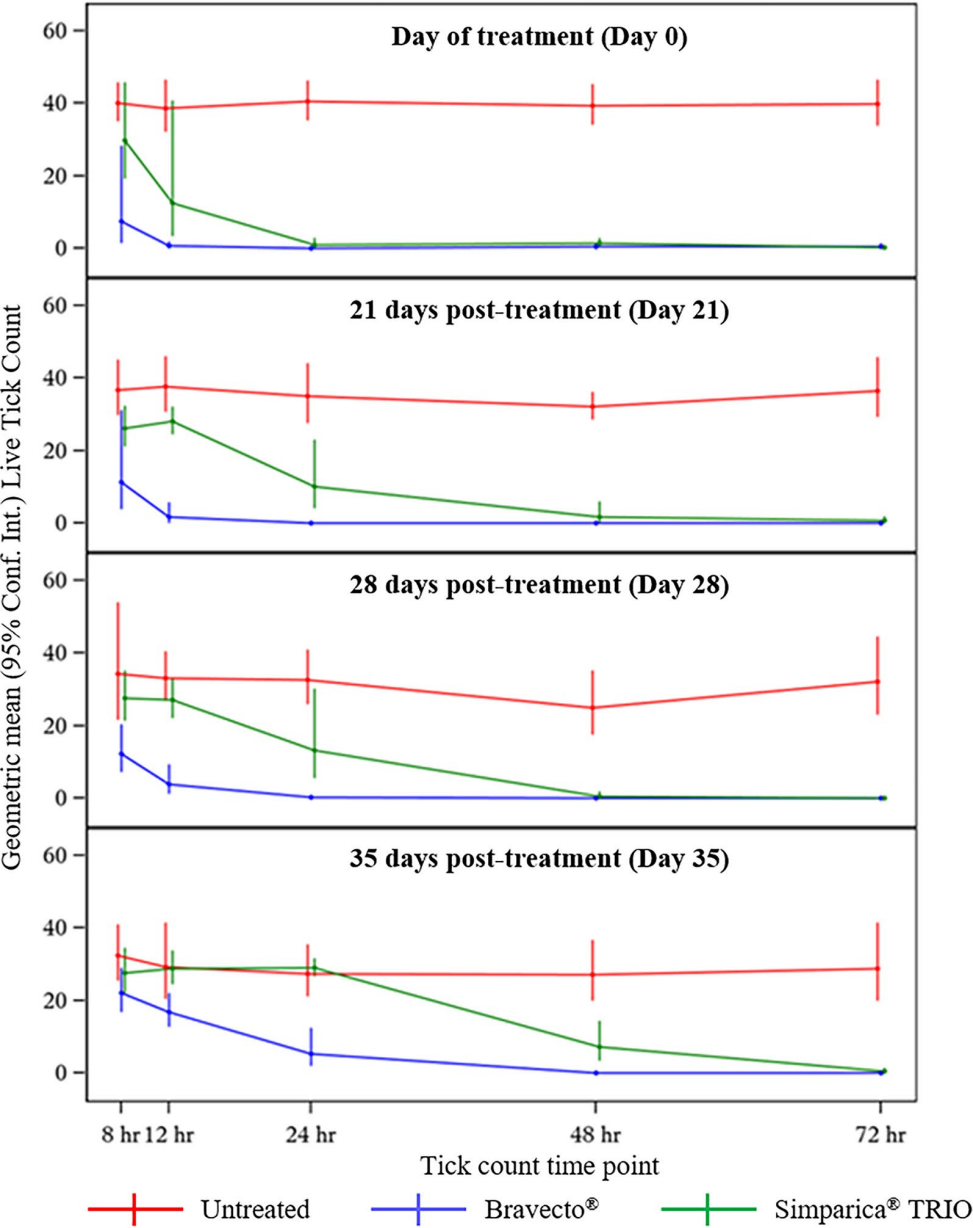
Tick kill rate of Bravecto® and Simparica TRIO® against adult *Amblyomma americanum* post-treatment and post-reinfestation. Live tick counts were conducted on dogs treated with Bravecto® or Simparica TRIO® or untreated at 8, 12, 24, 48, and 72 h post-treatment (day 0) and post-reinfestation on days 21, 28, and 35 post-treatment. Geometric mean (95% confidence interval) live tick counts are presented

Untreated control dogs maintained adequate infestations [10] throughout the entire study (Table 1). Changes in live tick counts after treatment or re-infestation are shown in Fig. 1. Compared with untreated dogs, significant control was first observed for Bravecto®-treated dogs by 8 h post-treatment (*P* = 0.020) and 24 h post-treatment for Simparica TRIO®-treated dogs (*P < *0.001) (Table 2). At 12 h post-treatment, the efficacy of Bravecto® and Simparica TRIO® was 98.0% and 67.4%, respectively. By 24 h post-treatment, the efficacy of Bravecto® and Simparica TRIO® was 99.8% and 97.7%, respectively (Table 2). Existing *A. americanum* infestations were controlled significantly more quickly for Bravecto® compared with Simparica TRIO®-treated dogs (Table 2).

When comparing the residual speed of tick kill against new tick infestation challenges at 21, 28, and 35 days post-treatment, Bravecto®-treated dogs had significantly fewer ticks at both the 12- and 24-h tick counts compared with both untreated and Simparica TRIO®-treated dogs (Table 2, Fig. 1). At reinfestation challenge 21 days post-treatment, tick control efficacy at 12 and 24 h post-infestation was 95.3% and 99.7% for Bravecto®-treated dogs and 25.5% and 70.9% for Simparica TRIO®-treated dogs, respectively (Table 2). By 72 h post-infestation, tick control efficacy for Bravecto®-treated and Simparica TRIO®-treated dogs was 100% and 97.9%, respectively (Table 2).

When dogs were reinfested with ticks on day 28, Bravecto®-treated dogs had significantly fewer ticks than Simparica TRIO®-treated dogs at 8, 12, and 24 h post-infestation (Table 2, Fig. 1). Efficacy at 12 h post-infestation was significantly higher for Bravecto®-treated compared with Simparica TRIO®-treated dogs (87.9% versus 18.3%, *P* < 0.001) (Table 2). By 24 h post-infestation, efficacy remained significantly greater for Bravecto®-treated compared with Simparica TRIO®-treated dogs (99.2% versus 59.3%, *P* < 0.001) (Table 2). By 48 h post-infestation, efficacy was 99.6% and 97.5% for Bravecto® and Simparica TRIO® treatment groups, respectively (Table 2).

When dogs were reinfested with ticks 35 days post-treatment, Bravecto®-treated dogs had significantly fewer ticks than Simparica TRIO®-treated dogs at 8, 12, 24, and 48 h post-infestation (Table 2, Fig. 1). Efficacy at 12 h post-infestation was significantly higher for Bravecto®-treated compared with Simparica TRIO®-treated dogs (42.2% versus 1.4%, *P* = 0.004) (Table 2). By 24 h post-infestation, efficacy remained significantly greater for Bravecto®- compared with Simparica TRIO®-treated dogs (80.6% versus 0.0%, *P* = 0.002) (Table 2). At 48 h post-infestation, tick control efficacy was still significantly greater for Bravecto®-treated compared with Simparica TRIO®-treated dogs (99.5% versus 73.1%, *P* < 0.001). By 72 h post-infestation, efficacy was 99.3% and 97.8% for Bravecto® and Simparica TRIO® treatment groups, respectively (Table 2).

### Tick-free dogs

When dogs were reinfested on day 21, 87.5% and 100% of dogs in the Bravecto® treatment group were tick free (i.e., free of live ticks) at 24 and 72 h post-infestation, respectively (Table 1). In contrast, 0% and 50% of the dogs in the Simparica TRIO® group were tick free at 24 and 72 h post-infestation, respectively (Table 1). Significantly more dogs were tick free by 24 h post-infestation in the Bravecto® treatment group compared with the Simparica TRIO® treatment group (*P* = 0.001) (Table 1).

Following the day 28 infestation, 75% and 100% of dogs in the Bravecto® treatment group were tick free by 24 and 72 h post-infestation, respectively. In contrast, 0% and 75% of dogs in the Simparica TRIO® group were tick free at 24 and 72 h post-infestation, respectively (Table 1). Significantly more dogs were tick free by 24 h post-infestation in the Bravecto® treatment group compared with the Simparica TRIO® treatment group (*P* = 0.007) (Table 1).

When dogs were reinfested on day 35, significantly more dogs in the Bravecto® treatment group were tick free compared with the Simparica TRIO® treatment group at 48 h post-infestation (87.5% versus 0%, *P* = 0.001) (Table 1).

## Discussion

When a dog is presented with attached ticks, pet owners want to know how quickly after treatment those attached ticks will be killed (initial speed of tick kill). Previously, Bravecto® was demonstrated to control *I. scapularis* tick infestations significantly more quickly than Simparica TRIO® for dogs provided a single dose of either product [9]. However, *I. scapularis* is generally regarded as an ‘easier’ tick to kill compared with *A. americanum*. Because *A. americanum* is a dominant and medically important tick species in a large portion of the USA, and is recognized as a challenging tick to control, the objective of the present study was to evaluate how quickly this tick species was controlled on dogs treated with a single dose of Bravecto® or Simparica TRIO®. In this study, the efficacy of a single oral dose of Bravecto® to treat an existing *A. americanum* infestation was 81.6% and 98.0% within 8 and 12 h of treatment, respectively, compared with 25.6% and 67.4% at those same timepoints for Simparica TRIO®-treated dogs (Table 2). The observed tick speed-of-kill was significantly faster for the Bravecto® treatment group than for the Simparica TRIO® treatment group (Table 2). The initial speed of *A. americanum* kill observed in this study for Bravecto®-treated dogs was more pronounced than in a previous study by Six et al. that observed 45.0%, 53.4%, and 97.8% *A. americanum* control within 8, 12, and 24 h of treatment, respectively [6]. The efficacy of Simparica TRIO® in the present study at 48 h post-treatment (96.4%) and 72 h post-treatment (99.3%) (Table 2) are in agreement with a previous study by Kryda et al. that observed 100% efficacy of Bertek Inc.-sourced *A. americanum* by 48 h post-treatment and 99.4% efficacy of Ecto Services-sourced *A. americanum* by 72 h post-treatment (efficacy for only one timepoint was reported per tick species and source in this study) [12]. The specific reason for the observed efficacy difference for Bravecto® in the Six et al. study at the earlier tick count timepoints is unknown, but one possibility could be the source and age of the ticks. Even ticks sourced from the same vendor may vary over time, as wild ticks are occasionally added to genetically refresh the tick colony. This difference highlights the need for caution when making comparisons across studies and the benefits of direct head-to-head comparative efficacy studies using the exact same experimental reagents and conditions.

The continued ability of an ectoparasiticide to prevent or minimize pathogen transmission is linked to its residual speed of tick kill upon reinfestation during the labeled treatment period (i.e., how quickly new tick infestations are controlled) [9]. It is commonly thought that ticks must be attached and feeding for about 24–48 h before pathogen transmission occurs; however, transmission times vary on the basis of the specific pathogen and other variables that may further influence transmission timing [13–15]. While there are numerous studies evaluating the transmission timing of *Borrelia burgdorferi* and *Anaplasma phagocytophilum* by *I. scapularis* [14–20], there are minimal data concerning pathogen transmission timing by *A. americanum*. For domestic cats, the transmission timing of *Cytauxzoon felis* by *A. americanum* was determined to occur between 36 h and 48 h post-infestation [21]. However, for *A. americanum*-associated pathogens that infect dogs, such as *Ehrlichia chaffeensis*, *E. ewingii*, *Rickettsia rickettsii*, and *Francisella tularensis* [1, 2, 22], pathogen transmission time studies are unavailable. Additional studies on the transmission-blocking efficacy of isoxazoline drugs against *A. americanum*-associated and other tick-borne pathogens are needed. Despite this lack of *A. americanum*-associated pathogen transmission timing data, extrapolation from other tick species that transmit pathogens to dogs support the need to kill ticks as quickly as possible to minimize pathogen transmission and disease risk [13].

Upon new *A. americanum* infestation challenges 21, 28, and 35 days post-treatment, significant tick control began for dogs in the Bravecto® treatment group by 8 h post-infestation on days 21, 28, and 35 compared with dogs in the untreated group (68.9%, 64.4%, and 31.5%, respectively) (Table 2). The efficacy of Bravecto® surpassed 90% by 24 h post-infestation on day 21 (99.7%) and day 28 (99.2%), and by 48 h on day 35 (99.5%) (Table 2). At 21, 28, and 35 days post-treatment, Bravecto®-treated dogs had significantly fewer live ticks than Simparica TRIO®-treated dogs at 12- and 24-h post-infestation timepoints (Table 2, Fig. 1).

This study was conducted to compare the speed of tick kill for *A. americanum*-infested dogs provided a single dose of Bravecto® or Simparica TRIO®. Of note, the Bravecto® product used in this study was the extended duration Bravecto® Chew product, which has an 8-week label versus Simparica TRIO®, which has a monthly label for *A. americanum*. An additional infestation challenge 35 days post-treatment was included to indicate how speed of tick kill may wane when a product is not readministered as scheduled and highlight the benefits of an extended duration tick control product. On the basis of sales records, lack of compliant readministration of tick control products by pet owners is common, with gaps in coverage worse for monthly dosed compared with extended duration tick control products [23]. In this study, while Simparica TRIO® had a slower speed of tick kill compared with Bravecto® at 8, 12, 24, and 48 h post-infestation on day 35, efficacy at 72 h was similar for both products. Efficacy at 72 h post-day 35 infestation for Bravecto® and Simparica TRIO® was 99.3% and 97.8%, respectively. The efficacy found for Simparica TRIO® in this study at 72 h post-day 35 infestation was similar to the efficacy reported for this product (99.3%) at this similar timepoint in a previous study where dogs were infested 33 days post-treatment and ticks counted on day 36 (72 h post-infestation) [12].

Although ≥ 90% tick control within a specified amount of time is required for a label indication for a given tick species, pet owners do not want to see any live ticks on their pets. In this study, a single Bravecto® treatment produced more tick-free dogs by 24 h post-infestation on days 21 and 28 post-treatment, with 87.5% (7/8) and 75% (6/8) dogs tick free, compared with 0% (0/8; *P* = 0.001) and 0% (0/8; *P* = 0.007) of dogs treated with Simparica TRIO® (Table 1). Finally, when dogs were reinfested 35 days post-treatment, 87.5% (7/8), of the Bravecto®-treated dogs were tick free, whereas none of the Simparica TRIO®-treated dogs were tick free by 48 h post-infestation (Table 1).

## Conclusions

Dogs treated with a single oral dose of Bravecto® (oral fluralaner) had an existing adult *Amblyomma americanum* tick infestation controlled by 98% by 12 h post-treatment compared with 67.4% control at this same timepoint for dogs treated with a single oral dose of Simparica TRIO®. Further, new *A. americanum* infestations at 21, 28, and 35 days post-treatment were controlled significantly more quickly for Bravecto®-treated dogs compared with Simparica TRIO®-treated dogs. Consistent with label indications, a single oral dose of Bravecto® or Simparica TRIO® effectively reduced live adult *A. americanum* by ≥ 97.9% by 72 h post-treatment and by 72 h post-infestations occurring 21 and 28 days post-treatment. At 21 and 28 days post-treatment, significantly more dogs treated with Bravecto® compared with Simparica TRIO® were tick free by 24 h post-infestation. To reduce the risk of tick-borne pathogen transmission to dogs, use of a tick control product that rapidly kills ticks is important.

## Data Availability

No datasets were generated or analysed during the current study.
